# How knowledge translation interventions involving health policymakers are evaluated: a scoping review protocol.

**DOI:** 10.12688/hrbopenres.14338.1

**Published:** 2026-03-28

**Authors:** Amelia Phillips, Medeina Macenaite, Molly Byrne, Shaun Treweek, Elaine Toomey

**Affiliations:** 1 School of Nursing and Midwifery, University of Galway, Galway, County Galway, H91 TK33, Ireland; 2 School of Health Sciences, University of Galway, Galway, County Galway, H91 TK33, Ireland; 3 School of Psychology, University of Galway, Galway, County Galway, H91 TK33, Ireland; 4 School of Medicine, Medical Sciences and Nutrition, University of Aberdeen, Aberdeen, Scotland, AB24 3FX, UK

**Keywords:** Knowledge translation; Knowledge translation interventions; Health policymaking; Health policymakers, Evaluation; Complex interventions; Scoping review; Evidence-informed policy; Evidence-informed decision-making.

## Abstract

**Background:**

Knowledge translation (KT) interventions are widely used to support the use of research evidence in health policymaking. These interventions vary in form, scope, and complexity, and are evaluated using a broad range of methodological approaches. Despite sustained attention to evidence-informed policymaking, there has been no comprehensive synthesis describing how KT interventions involving policymakers are evaluated, or how evaluation approaches are applied across different intervention types and policy contexts.

**Objectives:**

This scoping review aims to map and describe the evaluation methods used to evaluate KT interventions involving health policymakers. Specifically, the review seeks to (1) identify and categorise evaluation designs and methods used across KT intervention types; (2) describe the frameworks, concepts, and rationales reported to inform evaluation design choices; (3) examine how evaluation approaches are applied in relation to key intervention characteristics, including intervention type and complexity; and (4) summarise methodological characteristics and gaps in the existing evidence base.

**Methods:**

The review is conducted following established scoping review methodology and is reported in accordance with PRISMA-ScR guidance. Searches were conducted in major bibliographic databases and relevant grey literature sources. Eligible studies published in English from 2000 onwards evaluated a KT intervention involving health policymakers in health or public health contexts. Two reviewers independently screened records and extracted data using a piloted charting form. Evaluation approaches are being mapped descriptively across KT intervention types, with narrative synthesis used to identify recurring methodological patterns and gaps. Methodological characteristics are being examined descriptively using the Mixed Methods Appraisal Tool.

**Dissemination:**

Findings will be published in an open-access peer-reviewed journal and disseminated through conference presentations and plain-language summaries to support learning and transparency in the evaluation of KT interventions for policymaking.

## Background

Health policymakers play a central role in shaping health systems through policy design, resource allocation, and the governance of public service. ^1^ Strengthening the use of research evidence in policymaking is widely recognised as an important contributor to effective, efficient, and responsive health systems. ^2^ Despite sustained attention to evidence-informed policymaking, a gap persists between the production of research evidence and its use in policy decisions.

Knowledge translation (KT) refers to the dynamic and iterative processes through which research evidence is synthesised, disseminated, exchanged, and applied to inform decision-making. ^3^ KT interventions operationalise these processes and encompass a wide range of strategies, including dissemination tools, training and capacity-building initiatives, linkage and exchange mechanisms, knowledge brokering, policy dialogues, and embedded researcher programmes. These interventions differ substantially in their aims, mechanisms, and degrees of complexity.

KT interventions are commonly described using typologies such as push, pull, and exchange strategies. ^4^ Push strategies focus on the dissemination of evidence to policymakers, pull strategies aim to strengthen policymakers’ capacity to access and use evidence, and exchange strategies emphasise interaction and co-production between researchers and policymakers. Because these strategies operate through different causal pathways, they raise distinct evaluation challenges and may warrant different methodological approaches (see Table 1).

**Table 1.** Conceptual overview of knowledge translation (KT) intervention types and commonly reported evaluation approaches.

KT intervention type	Push, pull, exchange strategy	Description	Typical objectives of intervention	Potentially suitable evaluation designs	Accompanying rationale for evaluation
Dissemination/Communication (e.g., evidence briefs, tailored summaries, newsletters)	Push	Proactive provision of synthesised research in accessible formats to targeted policymakers. ^12^	Increase awareness, understanding, and intentions to use evidence.	RCT/cluster RCT; quasi-experimental (matched/stepped); before–after (pilots); ITS (when effects develop over time).	Discrete exposure with proximal outcomes makes randomisation or strong quasi-experimental designs feasible. Where randomisation is not possible, controlled before–after and ITS are recommended for causal inference in policy or health system interventions. Designs should align with complexity and context. ^11^
Capacity Building/Training for Policymakers	Push/Exchange	Workshops or coaching aimed at developing skills in appraisal, synthesis, and application. ^13^	Improve capability, confidence, and behaviours relating to evidence use.	RCT/cluster RCT; quasi-experimental; before–after with objective skills tests.	Training evaluation frameworks justify pre–post learning and behaviour measures, complemented by experimental or quasi-experimental tests where feasible. Design should reflect underlying mechanisms and context. ^14– 16^
Linkage & Exchange (Researcher–Policymaker Partnerships)	Exchange	Ongoing relational platforms for joint problem-framing, knowledge exchange, and co-production. ^17^	Strengthen relationships and integrate evidence into policy processes.	Mixed methods; realist evaluation; contribution analysis; case study.	Outcomes are emergent and context dependent and difficult to measure. Partnership theory and realist/contribution approaches explain “what works, for whom, in which contexts” when counterfactuals are weak. ^17, 18^
Knowledge Brokering/Intermediary Roles	Pull	Dedicated roles or teams mediating between research and policy (curation, translation, matchmaking). ^19^	Enable timely access to evidence, reframe problems, and support decisions.	Realist evaluation; contribution analysis; case study; developmental evaluation; mixed methods.	Work is heterogeneous and relational, so emphasis is on mechanisms and contribution rather than purely outcomes. Realist and contribution analysis provide credible causal accounts without strict counterfactuals. ^18, 20^
Policy Dialogue/Deliberative Forum	Exchange	Structured, facilitated deliberation on evidence-informed options. ^21^	Improve deliberative quality, shape agendas, and catalyse commitments.	Before–after (intentions/deliberative quality); mixed methods; case study; contribution analysis.	Dialogues contribute to proximal outcomes (e.g., knowledge, intentions) suitable for pre–post designs, while downstream influence is assessed via case studies, mixed methods, and contribution analysis. ^22, 23^
Embedded Researcher/Fellowship in Government	Exchange	Researcher(s) placed inside policy organisations to co-produce and support evidence use. ^24^	Build organisational capacity, change routines, and produce policy-relevant outputs.	Realist evaluation; contribution analysis; longitudinal case study; mixed methods; QCA (comparative cases).	Organisational and system change is context-rich and long-term. Theory-driven and case-comparative designs needed to investigate mechanisms and configurations across sites. ^25– 27^
Audit & Feedback to Policymakers	Push/Pull	Regular performance or evidence feedback provided to governance units. ^28^	Improve compliance, implementation, and adoption.	Cluster RCT; ITS; quasi-experimental.	Strong trial evidence supports RCTs for audit and feedback. Where randomisation is infeasible, ITS is a robust causal design for policy or system outcomes. EPOC endorses these designs for system-level interventions. ^29, 30^
Evidence Service/Portal (Including Rapid Response)	Pull	On-demand and/or self-serve access to curated syntheses and rapid response outputs. ^31^	Increase timely access and/or reduce time taken to utilise evidence to inform decisions.	Quasi-experimental (e.g., stepped adoption); before–after; mixed methods.	Evidence services primarily affect timeliness and access, making comparisons between adopters and non-adopters or pre/post use feasible. Design guidance recommends combining utilisation metrics with qualitative assessment of decision influence. ^31^
Communities of Practice/Networks	Exchange	Peer networks enabling mutual learning on policy-relevant topics. ^32^	Enhance mutual learning, diffusion of practices, and informal evidence use.	Mixed methods; case study; Social Network Analysis (SNA); realist evaluation (where mechanisms hypothesised).	Network interventions target relationships and brokerage. SNA captures network structure and ties, while qualitative and case designs explain governance and effectiveness. ^32– 34^
Multi-Component KT Programme	Any	Bundles of interventions (e.g., briefs and dialogue, training and brokering). ^35^	Sequential change from awareness to adoption to sustainment.	Mixed methods; realist evaluation; contribution analysis; ITS (for system-level trends).	High complexity and multiple pathways require integrated designs. Guidance recommends aligning design to complexity and using configurational methods to identify effective combinations. ^11^

Table 1 Summarises key KT intervention types involving policymakers and the evaluation approaches most frequently described in the KT and implementation science literature. The associations presented are informed by established KT typologies, methodological guidance on evaluating complex interventions, and illustrative examples from prior studies. This reflects a widely accepted principle that evaluation approaches should align with intervention characteristics and complexity. The table is intended as a heuristic framework to support analytic mapping and comparative synthesis in this review, rather than as a prescriptive guide or benchmark for evaluating individual studies. *ChatGPT* 4.1 was used to format the content and design this table. ^36^

Although a growing body of literature evaluates KT interventions involving policymakers ^5, 6^ existing studies vary widely in their evaluation designs, outcome measures, and use of theoretical or conceptual frameworks. Previous reviews and methodological analyses highlight variation in how evaluation approaches are selected, described, and reported. ^7, 8^

Guidance on the evaluation of complex interventions, such as KT interventions, ^3^ consistently emphasises that evaluation approaches should be aligned with the nature, complexity, and intended function of the intervention being evaluated, rather than privileging any single methodological design. ^9– 11^

Despite this guidance, there is currently no comprehensive synthesis describing how evaluation approaches are being applied across different types of KT interventions involving health policymakers, or how authors justify their methodological choices. This limits the ability of researchers, funders, and policymakers to interpret existing evaluations, compare approaches across contexts.

A scoping review is therefore warranted to systematically describe how and why particular approaches are selected, and identify areas where evidence is limited or unevenly distributed.

### Objectives

The overarching aim of this scoping review is to map and describe how knowledge translation (KT) interventions involving health policymakers are evaluated across health and public health contexts. The review seeks to characterise evaluation approaches and their application, rather than to assess intervention effectiveness or judge the quality of individual studies.

The primary objective is to identify and categorise the evaluation designs and methods applied to KT interventions involving policymakers.

The secondary objectives are to:

1. Describe the frameworks, concepts, or rationales reported by authors to inform their choice of evaluation approach.
2. Examine how evaluation approaches are applied in relation to key intervention characteristics, including intervention type, complexity, intended outcomes, and policy context.
3. Summarise the methodological characteristics of evaluations of KT interventions involving policymakers, including study designs and data collection methods.
4. Identify gaps and areas of limited coverage in the existing literature to inform future research and evaluation practice.

### Review questions

1. What evaluation designs and methods are used to evaluate KT interventions involving health policymakers?
2. What frameworks, theories, or rationales are reported to inform the choice of evaluation approach?
3. How are evaluation approaches applied across different KT intervention types and policy contexts?
4. What gaps are evident in the literature evaluating KT interventions involving policymakers?

## Methods

### Design and reporting standards

This scoping review protocol has been developed in accordance with the methodological framework originally proposed by Arksey and O’Malley, ^37^ with additional guidance from the Joanna Briggs Institute (JBI) Manual for Evidence Synthesis. The review will be reported in line with the Preferred Reporting Items for Systematic Reviews and Meta-Analyses extension for Scoping Reviews (PRISMA-ScR). The protocol will be prospectively registered on the Open Science Framework.

The purpose of this scoping review is to map and describe how KT interventions involving health policymakers are evaluated, rather than to assess intervention effectiveness or rank study quality.

### Eligibility criteria

Eligibility criteria were developed using the Population–Concept–Context (PCC) framework recommended for scoping reviews.

*Population*

The review will include studies evaluating KT interventions that involve health policymakers or policy-level decision-makers. Policymakers are defined as individuals with formal authority, advisory responsibility, or recognised influence over health or public health policy processes. ^1, 38^ This includes elected officials, senior civil servants, policy advisors within ministries or departments of health, senior managers in statutory health agencies, and appointed members of public health boards. Studies in which the term “policymaker” is used loosely will be assessed on a case-by-case basis and included only where a clear policy-level role can be identified. Where study populations are mixed, only studies reporting extractable data specific to policymakers will be included.

*Concept*

The concept of interest is the evaluation of KT interventions. Eligible studies must include an explicit evaluation component examining processes, outcomes, or impacts of a KT intervention. Evaluations may be qualitative, quantitative, or mixed-methods, including theory-driven or realist approaches. Editorials, commentaries, protocols without results, and purely conceptual papers will be excluded.

*Context*

Eligible contexts include health and public health policymaking organisations at local, regional, national, or international levels. These may include government ministries, statutory agencies, regional health authorities, and international organisations engaged in health policy. Studies conducted exclusively in clinical, academic, or service-delivery settings will be excluded unless they explicitly relate to policy-level decision-making.

### Timeframe and language

Only studies published in English from the year 2000 onwards will be included, reflecting the development of contemporary KT theory and evaluation practice.

### Information sources

The review will search major biomedical, social science, and interdisciplinary databases, including Scopus, MEDLINE, Embase, CINAHL, Web of Science, PsycINFO, and Global Health. To complement peer-reviewed literature, grey literature sources will be searched systematically, including relevant government repositories and organisational websites (e.g. EVIPNet, OECD). Backward and forward citation tracking will be undertaken for included studies.

### Search strategy

An initial search strategy was developed for Scopus and refined iteratively in consultation with the research team. Following piloting, the strategy was adapted for other databases using appropriate keywords and controlled vocabulary. Corresponding authors may be contacted to clarify missing or unclear information where necessary.

### Study selection

All identified records will be imported into Rayyan, review management software and de-duplicated prior to screening. Two reviewers (AP and MM) independently screened titles and abstracts against the eligibility criteria, followed by full-text screening of potentially relevant studies. Discrepancies were resolved through discussion or consultation with a third reviewer. Reasons for exclusion at full-text stage will be recorded and reported in the final review using a PRISMA-ScR flow diagram.

### Data charting

A data charting form will be used to extract information systematically from included studies. Extracted data will include publication characteristics (year, country), policy context, KT intervention type, evaluation design, data collection methods, outcomes assessed, and any frameworks or theories referenced by study authors. Information relating to intervention logic, evaluation focus, timeframe, and contextual considerations will be extracted where reported to support descriptive synthesis. Data charting will first be piloted by two reviewers for a subset of studies to ensure consistency, with refinements made as required. Data charting will then be conducted by AP with MM charting a random ten percent of studies.

### Analytic approach: examining how KT interventions are evaluated

Following completion of data charting, evaluation designs and methods will be synthesised descriptively to examine how knowledge translation (KT) interventions involving health policymakers are evaluated across health and public health contexts. Analysis will focus on identifying patterns, variation, and gaps in current evaluation practice.

Specifically, the analysis will consider:

• How evaluation designs and methods are applied across different KT intervention types (push, pull, exchange, and multi-component).
• The types of evaluation focus reported (process, outcome, or both) and the nature of outcomes assessed, including their proximity to stated intervention aims.
• The apparent alignment between original KT intervention characteristics and subsequent evaluation design choices, as reflected in reported rationales for design selection.
• The temporal framing of evaluations, including timing and duration of data collection in relation to the intervention.
• The extent to which contextual factors and mechanisms through which interventions operate are described or examined by study authors.

Findings will be synthesised narratively and presented by KT intervention type to highlight recurring methodological tendencies, areas of variation, and aspects of evaluation practice that appear under-represented or inconsistently reported. No formal assessment of effectiveness, causal attribution, or study-level appropriateness will be undertaken.

This analytic approach is intended to support reflection on current evaluation practice and to provide an empirical foundation for future methodological development, rather than to judge the quality or performance of individual studies.

### Critical appraisal

The methodological characteristics of included studies will be examined using the Mixed Methods Appraisal Tool (MMAT), 2018 version. ^39^ The MMAT will be applied descriptively to provide contextual information on study designs and reporting practices. MMAT findings will not be used to exclude studies or to generate composite quality scores but will be reported narratively to support interpretation of the evidence base.

### Synthesis of results

Results will be synthesised descriptively using tables and narrative summary. Evaluation approaches will be mapped across KT intervention types to illustyerate how different designs are used in practice. Patterns in evaluation focus, methodological approaches, and reporting characteristics will be highlighted in line with the objectives of the scoping review.

## Discussion

This scoping review will provide a comprehensive overview of how knowledge translation (KT) interventions involving health policymakers are evaluated across health and public health contexts. By systematically mapping evaluation designs, methods, and reported rationales, the review will offer insight into current methodological practices rather than drawing conclusions about intervention effectiveness.

The review is expected to highlight diversity in how KT interventions are evaluated, reflecting variation in intervention types, policy settings, and underlying assumptions about how interventions are expected to function. Evaluations of more discrete KT strategies, such as dissemination tools or training interventions, may rely predominantly on outcome-focused and experimental or quasi-experimental designs. In contrast, evaluations of more complex or relational interventions, including knowledge brokering, partnerships, or embedded researcher programmes, may more commonly employ qualitative or mixed methods approaches that emphasise context, mechanisms, and contribution. Mapping these patterns will clarify how evaluation approaches are currently applied in practice across different intervention categories.

By examining evaluation approaches in relation to intervention characteristics and evaluation design rationales, this review will contribute to ongoing methodological discussion about fit-for-purpose evaluation in KT and implementation research. Rather than judging individual studies, the review will identify areas where evaluation practices are applied consistently, where approaches vary widely, and where certain designs or methods appear under-represented or insufficiently reported.

Several limitations should be acknowledged. As with all scoping reviews, the analysis will be constrained by the quality and completeness of reporting in the included studies. Limited description of evaluation methods, intervention characteristics, or contextual factors may restrict the depth of synthesis. The review is restricted to English-language publications and studies published from 2000 onwards, which may exclude relevant work conducted in other languages or earlier periods.

## Ethics and dissemination

As this study involves secondary analysis of publicly available literature, ethical approval is not required. The review will nonetheless be conducted in accordance with principles of transparency, rigour, and responsible research practice.

Findings from the scoping review will be disseminated through multiple channels. The primary output will be publication in an open-access peer-reviewed journal. Results will also be presented at relevant academic and policy-focused conferences. To support accessibility and knowledge use, plain-language summaries will be developed for non-academic audiences, including policymakers and practitioners involved in KT activities. Where appropriate, findings may also be shared through policy-oriented briefings or research networks focused on evidence-informed policymaking.

## Conclusions

This scoping review will map and describe how KT interventions involving health policymakers are evaluated, addressing a gap in the current literature on evaluation practice in this field. By synthesising evaluation approaches across intervention types and policy contexts, the review will provide a structured overview of methodological trends and gaps.

The findings are intended to support methodological learning for researchers, evaluators, funders, and policymakers by clarifying how evaluation approaches are currently used and reported. In doing so, the review will contribute to strengthening the evidence base on KT evaluation and inform the design and reporting of future evaluations of KT interventions for policymaking.

## Data Availability

No datasets are associated with this article at the time of publication. This manuscript describes a protocol for a scoping review, and no results or extracted data are reported.
