# How knowledge translation interventions involving health policy decision makers are evaluated: a scoping review protocol.

**DOI:** 10.12688/hrbopenres.14338.2

**Published:** 2026-10-10

**Authors:** Amelia Phillips, Medeina Macenaite, Molly Byrne, Shaun Treweek, Elaine Toomey

**Affiliations:** 1 School of Nursing and Midwifery, University of Galway, Galway, County Galway, H91 TK33, Ireland; 2 School of Health Sciences, University of Galway, Galway, County Galway, H91 TK33, Ireland; 3 School of Psychology, University of Galway, Galway, County Galway, H91 TK33, Ireland; 4 School of Medicine, Medical Sciences and Nutrition, University of Aberdeen, Aberdeen, Scotland, AB24 3FX, UK

**Keywords:** Knowledge translation; Knowledge translation interventions; Health policymaking; Health policymakers, Evaluation; Complex interventions; Scoping review; Evidence-informed policy; Evidence-informed decision-making.

## Abstract

**Background:**

Knowledge translation (KT) interventions support the use of research evidence in health policymaking but vary substantially in form, complexity and evaluation. There is no comprehensive synthesis of how KT interventions involving health policy decision makers are evaluated across intervention types and policy contexts.

**Objectives:**

This scoping review aims to map and describe how KT interventions involving health policy decision makers are evaluated. It will identify evaluation approaches, designs and methods; describe theories, frameworks, programme logics and rationales informing evaluation choices; examine how evaluation characteristics vary by intervention type, complexity, intended outcomes, policy context and evaluation use; and identify gaps in evaluative coverage.

**Methods:**

This review will be conducted in accordance with established scoping review guidance and reported using PRISMA-ScR. Eligibility will be structured using the Population–Concept–Context framework. Studies published in English from 2000 onwards that empirically evaluate a KT intervention involving or directed towards health policy decision makers will be eligible. Evaluation characteristics will be examined using descriptive numerical mapping, Lemire’s Evaluation Metro Map as an organising and communication tool, and structured qualitative content analysis. The Mixed Methods Appraisal Tool will provide supplementary methodological context. The review will characterise evaluation practice rather than estimate intervention effectiveness or rank studies.

**Dissemination:**

Findings will be published in an open-access peer-reviewed journal and disseminated through conference presentations and plain-language summaries for academic and policy audiences.

## Background

Health policy decision makers play a central role in shaping health systems through policy development, implementation, resource allocation, regulation and governance. Strengthening the use of research evidence in policymaking is widely recognised as an important contributor to effective, efficient and responsive health systems. Despite sustained attention to evidence-informed policymaking, a gap persists between the production of research evidence and its use in policy decisions. ^1^ For the purposes of this review, health policy decision makers are individuals who hold a formal or substantive role in developing, influencing, advising on, commissioning, implementing or governing decisions concerning health policy, public health policy, health services or health systems. ^1^^,^^2^ This may include people working in government departments, public health agencies, statutory health organisations, regulatory bodies and other organisations with responsibility for health policy decisions. Individuals outside the health sector will be included only where their role involves identifiable decisions that directly affect health policy, population health or health-system organisation. Strengthening the use of research evidence in policymaking is widely recognised as an important contributor to effective, efficient, and responsive health systems. ^3^ Despite sustained attenton to evidence-informed policymaking, a gap persists between the production of research evidence and its use in policy decisions.

Knowledge translation (KT) refers to the dynamic and iterative processes through which research evidence is synthesised, disseminated, exchanged, and applied to inform decision-making. ^4^ KT interventions operationalise these processes and encompass a wide range of strategies, including dissemination tools, training and capacity-building initiatives, linkage and exchange mechanisms, knowledge brokering, policy dialogues, and embedded researcher programmes. These interventions differ substantially in their aims, mechanisms, and degrees of complexity.

KT interventions are commonly described using broad push, pull and exchange orientations. ^4^ Push strategies focus primarily on communicating or disseminating evidence to decision makers; pull strategies strengthen decision makers’ capacity or opportunities to access and use evidence; and exchange strategies emphasise interaction, relationship-building and co-production between researchers and decision makers. Interventions may combine more than one orientation. ^5^ Table 1 provides a conceptual overview and will inform descriptive categorisation of the KT interventions without being used as a rigid classification system.

**Table 1.** Conceptual overview of KT intervention orientations.

KT orientation	Illustrative interventions	How it seeks to support evidence use	Illustrative evaluation considerations
**Push**	Policy briefs, evidence summaries and other forms of targeted dissemination. ^6^	Makes research more accessible, relevant or timely to health policy decision makers. ^6^	Reach, clarity, accessibility, engagement, uptake and use of evidence products. Evaluation design should reflect the nature and complexity of the intervention and the outcomes of interest. ^6^^,^^7^
**Pull**	Training and capacity-building interventions, ^8^^,^^11^ evidence appraisal support, and responsive or rapid evidence services. ^12^	Strengthens demand for evidence and decision makers’ capacity to find, appraise and apply it. ^8^^,^^10^^,^^12^	Knowledge, skills, confidence, service responsiveness and subsequent use of evidence in health policy work. Training outcomes may be examined at learning and behavioural levels, while evidence services may also require assessment of timeliness, utilisation and influence on decision-making ^9^^,^^11^^,^^12^
**Exchange**	Policy dialogues, ^13^^,^^15^ knowledge brokering, ^16^^,^^17^ research–policy partnerships and co-production, ^18^ and other relational or participatory approaches.	Supports interaction, shared understanding, relationship-building and joint knowledge production between researchers and health policy decision makers. ^15^^,^^16^^,^^18^	Engagement, trust, mechanisms, context, contribution, partnership functioning and policy-process learning. Relational and context-dependent interventions may require mixed-method, theory-informed or contribution-focused evaluation ^14^^,^^15^^,^^17^^–^^19^
**Combined or multicomponent**	Organisational KT programmes and interventions combining dissemination, capacity-building and exchange activities. ^20^	Acts through multiple mechanisms and at multiple levels of the health policy system.	Programme theory, implementation, adaptation, system change, contribution and outcomes over time. Evaluation should account for interacting components, mechanisms and contextual influences rather than assuming a single linear pathway to effect. ^7^^,^^20^

**Note.** The table supports description of the KT intervention, whereas the Evaluation Metro Map supports communication of different dimensions of its evaluation. Interventions may span more than one orientation.

Although a growing body of literature evaluates KT interventions involving health policymakers ^21, 22^ existing studies vary widely in their evaluation designs, outcome measures, and use of theoretical or conceptual frameworks. Previous reviews and methodological analyses highlight variation in how evaluation approaches are selected, described, and reported. ^23, 24^

Guidance on the evaluation of complex interventions, including KT interventions, ^4^ emphasises that evaluation should be aligned with the nature, complexity and intended function of the intervention rather than privileging any single methodological design. ^7^^,^^25^^,^^26^ Lemire’s Evaluation Metro Map provides a shared language for separating dimensions that are often conflated, including intended evaluation use, theoretical orientation and design ^27^ In this review, the Metro Map will be used as an organising and communication tool, with particular attention to the use, theory and design lines. It will not be used as a quality-assessment or ranking tool. Despite this guidance, there is currently no comprehensive synthesis describing how evaluation approaches are being applied across different types of KT interventions involving health policymakers, or how authors justify their methodological choices. This limits the ability of researchers, funders, and health policymakers to interpret existing evaluations, compare approaches across contexts.

Despite this guidance, there is currently no comprehensive synthesis describing how evaluation approaches are being applied across different types of KT interventions involving health policymakers, or how authors justify their methodological choices. This limits the ability of researchers, funders, and health policymakers to interpret existing evaluations, compare approaches across contexts.

A scoping review is therefore warranted because the purpose is to map and characterise a broad and heterogeneous evidence base concerning evaluation purpose, theory, approach, design, methods and outcomes, rather than to estimate intervention effectiveness, compare a narrowly specified set of interventions or produce a pooled estimate. This design is suited to identifying how evaluation has been undertaken and where evidence or methodological reporting is limited. ^28^^,^^29^

### Objectives

The overarching aim of this scoping review is to map and describe how KT interventions involving health policy decision makers are evaluated across health and public health contexts. The review seeks to characterise evaluation approaches and their application rather than assess intervention effectiveness or rank individual studies.

The primary objective is to identify and categorise the evaluation approaches, designs and methods applied to KT interventions involving health policy decision makers.

The secondary objectives are to:

1. Describe the theories, frameworks, programme logics or rationales reported by authors as informing evaluation choices.
2. Examine how evaluation characteristics are applied in relation to KT intervention type, complexity, intended outcomes, policy context and intended evaluation use.
3. Summarise the outcomes, timing, contextual attention and methodological characteristics of the evaluations.
4. Identify gaps and areas of limited or inconsistent evaluative coverage to inform future research and evaluation practice.

### Review questions

1. What evaluation approaches, designs and methods are used to evaluate KT interventions involving health policy decision makers?
2. What theories, frameworks, programme logics or rationales are reported to inform the evaluation?
3. How do evaluation use, theory, design, methods and outcomes vary across different KT intervention types and policy contexts?
4. What gaps or areas of limited reporting are evident in the literature evaluating KT interventions involving health policy decision makers?

## Methods

### Design and reporting standards

This scoping review protocol has been developed in accordance with the methodological framework originally proposed by Arksey and O’Malley, ^30^ with contemporary guidance on the conduct, extraction, analysis and presentation of scoping reviews. ^29^ The review will be reported in line with the Preferred Reporting Items for Systematic Reviews and Meta-Analyses extension for Scoping Reviews (PRISMA-ScR). ^31^ The protocol will be prospectively registered on the Open Science Framework.

A scoping review was selected because the purpose is to identify and characterise the range of evaluation approaches, designs, methods and outcomes used across a heterogeneous body of KT interventions and policy settings. It is not intended to estimate intervention effectiveness, compare a narrowly specified intervention or produce a pooled effect estimate. The review questions therefore operate as related subquestions under one overarching mapping objective. ^28^

### Eligibility criteria

Eligibility criteria are structured using the Population–Concept–Context (PCC) framework because the review seeks to map and characterise a broad and heterogeneous evidence base rather than compare a narrowly specified intervention and outcome. ^29^ PCC corresponds directly to the review: the population is health policy decision makers; the concept is empirical evaluation of a KT intervention involving or directed towards them; and the context is health policy, health-service and health-system decision-making. This framework permits inclusion of diverse KT interventions, evaluation approaches, designs, methods and policy contexts.

*Population*

The review will include studies evaluating KT interventions that involve or are directed towards health policy decision makers, as defined in the Background. ^1^^,^^2^ This may include elected officials, senior civil servants, policy advisers within ministries or departments of health, senior managers in statutory health agencies, regulatory decision makers and appointed members of public health or health-system boards. Studies in which the term “policymaker” is used loosely will be assessed on a case-by-case basis and included only where a clear health policy decision-making role can be identified. Where study populations are mixed, only studies with identifiable or extractable data relating to health policy decision makers will be included.

*Concept*

The concept of interest is the empirical evaluation of a KT intervention intended to support the accessibility, exchange, interpretation or use of research evidence by health policy decision makers. _4_ Eligible studies must include an identifiable empirical evaluative component examining implementation, processes, use, experience, outcomes, impacts, mechanisms or contribution. Evaluations may use qualitative, quantitative or mixed methods and may draw on named theory-driven approaches. Editorials, commentaries, protocols without results, purely conceptual papers and descriptive accounts of KT activity without empirical evaluation data will be excluded.

*Context*

Eligible contexts include health and public health policymaking at local, regional, national or international levels, including health-service and health-system decision-making. These may include government ministries, statutory agencies, regional health authorities, regulatory bodies and international organisations engaged in health policy. Studies conducted exclusively in clinical, patient-facing, educational, academic or service-delivery settings will be excluded unless the KT intervention explicitly relates to health policy or health-system decision-making.

### Timeframe and language

Studies published in English from 2000 to the date on which each database search was conducted were eligible. The English-language restriction is retained as a feasibility constraint. Machine-assisted translation was considered, but the review team did not have the language expertise required to verify detailed extraction of methodological descriptions, theoretical concepts and context-specific policy terminology across potentially eligible non-English studies. Because the review requires detailed classification of evaluation theory, rationale, design, methods, outcomes and policy context, the team retained the English-language restriction. The restriction may nevertheless reduce the geographical and linguistic breadth of the evidence base and will be acknowledged as a limitation.

### Information sources

Searches were conducted in PubMed, Scopus, APA PsycINFO and ScienceDirect between 13 and 28 October 2025. The four database searches yielded 6,261 records before deduplication: PubMed (n = 3,259), Scopus (n = 1,658), APA PsycINFO (n = 1,236) and ScienceDirect (n = 108). Backward citation chasing was undertaken using the included studies.

### Search strategy

The original search strategy was developed iteratively for the databases used in the review. It was not prospectively peer reviewed using the PRESS framework because of timing and resource constraints within the PhD programme. A retrospective PRESS assessment was subsequently undertaken by an information specialist at the University of Galway. ^32^ The assessment informed transparent reporting of the completed strategy and identified recommendations for future or updated searching, including database-platform choice, line-by-line reporting with hit counts, use of database-specific subject headings alongside free-text terms, spelling and proximity variants, evaluation terminology and consideration of overlap between ScienceDirect and Scopus. The retrospective assessment will not be represented as prospective validation of the completed searches.

The database-level results were as follows: ScienceDirect, n = 108; PubMed, n = 3,259; Scopus, n = 1,658; APA PsycINFO, n = 1,236; total before deduplication, n = 6,261. A comprehensive breakdown of the search strategy has been linked in the data availability section of this paper.

### Study selection

Records were imported into Rayyan for deduplication, screening and management of review decisions. ^33^ Two reviewers independently screened all titles and abstracts. At full-text stage, 20% of records were assessed by a second reviewer to support consistency. Disagreements and uncertainties were resolved through discussion and, where required, consultation with an independent researcher. Reasons for full-text exclusion were recorded and will be reported in the final review using a PRISMA-ScR flow diagram. ^31^

### Data charting

A structured data-charting form will be used to extract information systematically from included studies. The unit of analysis will be each included study. Data-charting items are grouped by domain in Table 2 to improve transparency and reproducibility. ^29^ Data charting will first be piloted by two reviewers on a subset of studies, with refinements made as required, before full charting is completed by AP with a second reviewer checking a sample for consistency.

**Table 2.** Data-charting framework.

Domain	Information to be charted
Study characteristics	Author, year, country/region, publication type, setting and study purpose.
Health policy decision-maker population	Role, organisational location, level of decision-making, sample characteristics, and sex and/or gender where reported.
KT intervention	Aim, components, mechanisms, duration, target group, broad push/pull/exchange orientation and degree of complexity or multicomponent delivery.
Policy context	Health or public health area, decision-making level, organisational context and wider health-system context.
Evaluation use and theoretical orientation	Evaluation purpose or intended use; programme theory, theory of change, logic model, realist logic, contribution logic or other theoretical/conceptual basis.
Evaluation approach	Named broader evaluation orientation where explicitly reported, such as realist, utilisation-focused, developmental or equity-focused evaluation; unclear or unreported approaches retained as such.
Evaluation design	Empirical structure, such as case study, randomised trial, quasi-experimental design, interrupted time series, before-and-after design, qualitative design or mixed-methods design.
Methods and analysis	Data-collection sources, sampling, timing, analytical methods and integration of qualitative and quantitative evidence where applicable.
Outcomes and evaluative focus	Process, implementation, engagement, capacity, evidence use, policy or system outcomes; timing and proximity to intervention aims.
Sex and gender reporting	Terminology used by authors; whether sex and/or gender are reported; whether findings are disaggregated or examined by sex/gender. Neither characteristic will be inferred.
Methodological limitations and reflections	Author-reported limitations, feasibility issues, contextual constraints, adaptations and reflections on evaluation choices.

Sex- and gender-related reporting will be handled in line with the SAGER guidelines. ^34^ The terminology used by study authors will be retained; sex and gender will not be treated as interchangeable; and neither characteristic will be inferred where it is not reported. Where findings are reported or examined by sex or gender this will be recorded. The review will also report the extent to which sex and gender are absent from evaluation reporting.

### Analytic approach: examining how KT interventions are evaluated

Analysis will proceed in three related stages. First, descriptive numerical summaries will map study characteristics, KT intervention types, health policy decision-maker populations, policy contexts, evaluation approaches and designs, data-collection methods, outcomes and other categorical variables. Counts will be interpreted as characteristics of the included evidence base rather than estimates of prevalence beyond that evidence base. ^29^

Second, Lemire’s Evaluation Metro Map will be used as an organising and communication tool. ^27^ The use line will capture the apparent purpose or intended use of the evaluation; the theory line will capture the extent to which programme theory, realist logic, contribution analysis, theory of change, logic models or other frameworks shape the evaluation; and the design line will capture the empirical structure. Information explicitly reported by study authors will be distinguished from cautious reviewer interpretation. A named evaluation approach will not be assigned from design alone, and unclear information will be retained as not reported or uncertain.

Third, structured qualitative content analysis ^35^ will be used to examine recurring relationships among intervention aims, mechanisms, complexity, intended outcomes, policy context and evaluation choices. ^29^ The analysis will consider evaluation focus and intended use, the nature and timing of outcomes, the proximity of outcomes to stated intervention aims, and how intervention characteristics are reflected in reported evaluation choices. Attention will also be given to author-reported rationales for evaluation choices and to the extent to which theory, programme logic, contextual factors and mechanisms are described or examined.

Patterns will be compared across broad push, pull, exchange and multicomponent KT orientations, while recognising that interventions may span more than one orientation. The purpose will be to identify recurring patterns, areas of variation and gaps in reporting or evaluative coverage. The analysis will not be used to determine intervention effectiveness, make causal attribution or rank evaluation designs.

Findings will be presented using descriptive tables, cross-tabulations where informative, study-level mapping and a narrative account of recurring patterns and gaps. ^29^ Table 1 will support description of the KT interventions, whereas the Evaluation Metro Map will support communication of the different dimensions of their evaluation. ^27^ These analytic lenses are complementary and neither will be used as a quality-ranking system.

This analytic approach is intended to support reflection on current evaluation practice and provide an empirical foundation for future methodological development. It is not intended to judge the effectiveness, methodological worth or performance of individual KT interventions or evaluations.

### Critical appraisal

The methodological characteristics of included studies will be examined using the Mixed Methods Appraisal Tool (MMAT), 2018 version. ^36^ The MMAT will be applied descriptively as supplementary methodological context. Findings will not be used to exclude studies, generate an overall numerical quality score or rank evaluation designs, but will support interpretation of the characteristics and reporting of the evidence base.

### Synthesis of results

Results will be presented in tables and narrative form aligned to the review questions. ^29^ Descriptive mapping will show the distribution of KT intervention orientations, evaluation approaches, designs, methods and outcomes; Metro Map-oriented summaries will distinguish intended use, theoretical orientation and design ^27^; and the structured content analysis will describe recurring relationships, variation and gaps. The synthesis will remain descriptive and interpretive rather than effectiveness-focused.

## Discussion

This scoping review will provide a comprehensive overview of how knowledge translation (KT) interventions involving health policy decision makers are evaluated across health and public health contexts. By systematically mapping evaluation approaches, designs, methods, intended uses and reported rationales, the review will offer insight into current methodological practices rather than drawing conclusions about intervention effectiveness.

The review is expected to highlight diversity in how KT interventions are evaluated, reflecting variation in intervention types, policy settings and assumptions about how interventions are expected to function. Mapping these characteristics will clarify whether particular evaluative purposes, theoretical orientations, designs or methods are concentrated in particular intervention categories and where relationships are unclear or inconsistently reported.

By examining evaluation approaches in relation to intervention characteristics, intended evaluation use and reported rationales, this review will contribute to methodological discussion about fit-for-purpose evaluation in KT and implementation research. Rather than judging individual studies, the review will identify areas where evaluation practices are reported consistently, where approaches vary and where aspects of evaluation appear under-represented or insufficiently described.

Several limitations should be acknowledged. As with all scoping reviews, the analysis will be constrained by the quality and completeness of reporting in the included studies. Limited description of evaluation methods, intervention characteristics, theory or contextual factors may restrict the depth of interpretation. The English-language restriction may reduce the geographical and linguistic breadth of the evidence base. Sex and gender may also be reported inconsistently or not at all. Where the Metro Map is used to orient studies, explicit author reporting will be distinguished from cautious reviewer interpretation to avoid over-classification.

## Ethics and dissemination

As this study involves secondary analysis of publicly available literature, ethical approval is not required. The review will nonetheless be conducted in accordance with principles of transparency, rigour, and responsible research practice.

No health policy decision makers are formal members of the review team. Health policy decision makers will be involved at the dissemination and knowledge-translation stage to support the accessibility and relevance of the review findings, and their involvement is planned across subsequent studies within the wider PhD programme.

Findings from the scoping review will be disseminated through multiple channels. The primary output will be publication in an open-access peer-reviewed journal. Results will also be presented at relevant academic and policy-focused conferences. To support accessibility and knowledge use, plain-language summaries will be developed for non-academic audiences, including policymakers and practitioners involved in KT activities. Where appropriate, findings may also be shared through policy-oriented briefings or research networks focused on evidence-informed policymaking.

## Conclusions

This scoping review will map and describe how KT interventions involving health policy decision makers are evaluated, addressing a gap in the current literature on evaluation practice in this field. By distinguishing evaluation purpose, theory, approach, design and methods across intervention types and policy contexts, the review will provide a structured overview of methodological patterns and gaps.

The findings are intended to support methodological learning for researchers, evaluators, funders and health policy decision makers by clarifying how evaluation approaches are used and reported. In doing so, the review will contribute to strengthening the evidence base on KT evaluation and inform the design and reporting of future evaluations intended to support health policymaking.

## Data Availability

No data are associated with this article. OSF: Full database search strategies for “How knowledge translation interventions involving health policy decision makers are evaluated: a scoping review protocol”. DOI: https://doi.org/10.17605/OSF.IO/5PKFG. This project contains the following extended data:
• Full search strategy for the scoping review. Data are available under the terms of the CC0 1.0 Public Domain Dedication.
