# Supplementary material for: How knowledge translation interventions involving health policy decision makers are evaluated: a scoping review protocol
Source: HRB Open Res. 2026 Oct 10;9:27. Originally published 2026 Mar 28. [Version 2] doi: 10.12688/hrbopenres.14338.2 (PMC13653105; doi:10.12688/hrbopenres.14338.2)
Supplement: Peer review history

# Reviewer report: Reviewer response for version 2

**DOI:** 10.21956/hrbopenres.16042.r58468

**Published:** 2026-10-16

**Authors:** Lupin Battersby

**Affiliations:** 1 Simon Fraser University, Burnaby, Canada

Nothing to change. And we approve.

Is the study design appropriate for the research question?

Yes

Is the rationale for, and objectives of, the study clearly described?

Yes

Are sufficient details of the methods provided to allow replication by others?

Partly

Are the datasets clearly presented in a useable and accessible format?

Not applicable

Reviewer Expertise:

NA

I confirm that I have read this submission and believe that I have an appropriate level of expertise to confirm that it is of an acceptable scientific standard.

# Reviewer report: Reviewer response for version 1

**DOI:** 10.21956/hrbopenres.15790.r54471

**Published:** 2026-10-09

**Authors:** Lupin Battersby, Alison J. Moore

**Affiliations:** 1 Simon Fraser University, Burnaby, Canada

Note, this review was completed with Alison Moore.

 This manuscript describes the protocol for a scoping review on evaluating knowledge translation (KT) interventions in health policy. This is an important and understudied area, and therefore, this scoping review will provide a meaningful contribution to the literature in this area. Here we provide some observations and critiques for consideration. 

 We noted that, while you will be conducting a more thorough review of the literature, there is recent or other related literature not included in the background that might be useful for framing and rationale of this scoping review to ensure you are building on what is known. Some examples of this include:  

- Sarkies M, Bowles K, Skinner E, Haas R, et al., 2017 ^1^
- Chriqui J, Asada Y, Smith N, Kroll-Desrosiers A, et al., 2023 ^2^

To ensure transparency and replicability of this study (and move question 3 from partly to yes), more information about the search string is required. Sharing a sample search string for one of your selected databases would be ideal, but if full search strings cannot be shared, then a list of search terms included in the study would be helpful for your reader. Given there is so much variation in terminology (acronyms, like terms, and alternative spellings) a discussion of which synonyms for knowledge translation (including knowledge mobilization, implementation and dissemination, knowledge transfer and exchange, etc.) were included in your study would be beneficial. Additionally, if you have not already connected with an academic librarian about your search strategy and protocol development, you may find it useful to do so. 

 While the draft is clearly written and easy to follow, we do recommend an overall copyedit and review for occasional typos and verb tense agreement. We echo reviewer 1 on being clear about your definitions, particularly with reference to evaluation (approach versus design), and defining policy makers earlier in the paper. We note there are citations missing for in text mentions of JBI and Prisma-ScR, and these should be included in the reference list (e.g., Tricco A, Lillie E, Zarin W, O'Brien K, et al., 2018 ^3^). Finally, the secondary objectives could likely be cut, as the review questions are clear and having the objectives and the questions is redundant. This would leave more space in the paper to define concepts, share the search string, and provide additional details as needed.  

 We look forward to reading the completed scoping review paper.

Is the study design appropriate for the research question?

Yes

Is the rationale for, and objectives of, the study clearly described?

Yes

Are sufficient details of the methods provided to allow replication by others?

Partly

Are the datasets clearly presented in a useable and accessible format?

Not applicable

Reviewer Expertise:

Lupin - knowledge mobilization, implementation science, health system, health policy; Alison - library and information science, scholarly communication, knowledge mobilization

We confirm that we have read this submission and believe that we have an appropriate level of expertise to confirm that it is of an acceptable scientific standard.

## References

[1] : The effectiveness of research implementation strategies for promoting evidence-informed policy and management decisions in healthcare: a systematic review. *Implementation Science* .2017;12(1) : 10.1186/s13012-017-0662-0 10.1186/s13012-017-0662-0 PMC568680629137659

[2] : Advancing the science of policy implementation: a call to action for the implementation science field. *Translational Behavioral Medicine* .2023;13(11) : 10.1093/tbm/ibad034820-825 10.1093/tbm/ibad034 PMC1063187337354558

[3] : PRISMA Extension for Scoping Reviews (PRISMA-ScR): Checklist and Explanation. *Annals of Internal Medicine* .2018;169(7) : 10.7326/M18-0850467-473 10.7326/M18-0850 30178033

# Reviewer report: Reviewer response for version 1

**DOI:** 10.21956/hrbopenres.15790.r54469

**Published:** 2026-05-20

**Authors:** Jacqui Cameron

**Affiliations:** 1 University of Wollongong, Wollongong, Australia

Thank you for the opportunity to review this well written and important protocol.

 Overall, the manuscript is well written and complies with scope for this journal.

 My suggestions for revisions are very minor.

**Abstract**

 Change the first sentence of the methods. “This review will be conducted…”

**Background**

 I am not sure of the inclusion of Table 1 here, could this be used for adding a valuable lens to the to the analysis (see comment below).

**Methods**

 Why did you choose PCC? Please explain.

 The data charting list is extensive, a table here may help the reader.

**Analysis**

 The analysis beyond data charting is vague, there are no references so it is not clear what method/methodology this additional analysis will follow apart from ‘synthesised descriptively’ and then ‘synthesised narratively’ – these need to be explained further.

**Overall comments**

 Overall, my concern only related to the scope, as it seems large for a scoping review. There are many RQ to answer and that may not be possible with a SR methodology. Is a systematic review rather than a scoping review warranted? Given there is expectation of several completed studies this may warrant consideration.

 There are sentences written in both past and present and future tense, which is a bit confusing, can you please check this and refine for future tense.

 There are many missing references, please check and fix. E.g. PRISMA-ScR, Rayyan etc

Is the study design appropriate for the research question?

Partly

Is the rationale for, and objectives of, the study clearly described?

Yes

Are sufficient details of the methods provided to allow replication by others?

Partly

Are the datasets clearly presented in a useable and accessible format?

Yes

Reviewer Expertise:

Systematic reviews, scoping reviews, KT, IKT

I confirm that I have read this submission and believe that I have an appropriate level of expertise to confirm that it is of an acceptable scientific standard, however I have significant reservations, as outlined above.

# Response

**Published:** 2026-07-15

**Authors:** Amelia Phillips

**Affiliations:** Nursing and Midwifery, University of Galway, Galway, County Galway, Ireland

We sincerely thank Dr Cameron for her careful review and encouraging assessment of the protocol. Her comments helped us improve the clarity and transparency of the methods, particularly the rationale for using PCC, the presentation of the data-charting framework, the description of the analysis, and the justification for retaining a scoping review design. We have revised the manuscript accordingly.

**General comments**

**Comment:**

*The manuscript is well written, important and within the scope of the journal. The suggested revisions are minor.*

**Response:**

 We thank the reviewer for the positive assessment and for the constructive recommendations provided.

**Abstract wording**

**Comment:**

*Change the first sentence of the Methods section of the abstract to “This review will be conducted…”.*

***Response:***

 This sentence has been amended as suggested.

**Position and analytical role of Table 1**

**Comment:**

*The inclusion of Table 1 in the Background is uncertain. It may provide a valuable analytical lens.*

**Response:**

 We agree that the purpose of Table 1 required clarification. The table will be retained as a conceptual overview of the forms of KT intervention potentially relevant to the review. As recommended by Reviewer 1, it has been reorganised around push, pull and exchange orientations.

 The table will also inform the descriptive categorisation of the included interventions during data charting and analysis. However, it will not be used as a rigid classification system, as interventions may incorporate more than one KT orientation.

 Its function is distinct from that of the Evaluation Metro Map. Table 1 will support the description of the KT interventions, whereas the Metro Map will support communication of the different dimensions of their evaluation. This distinction has been made explicit in the manuscript.

**Rationale for using PCC**

**Comment:**

*Please explain why the Population, Concept and Context framework was selected.*

**Response:**

 A rationale for using the PCC framework has been added. PCC was selected because the purpose of the review is to map and characterise a broad and heterogeneous body of evidence rather than evaluate the effectiveness of a narrowly defined intervention.

 The framework corresponds directly to the review question and eligibility criteria:

- Population: health policy decision makers;
- Concept: the evaluation of KT interventions involving or directed towards health policy decision makers; and
- Context: health policy, health service and health-system decision-making settings.

Unlike frameworks structured primarily around intervention comparisons and outcomes, PCC allows the review to include diverse forms of KT intervention, evaluation design, methodological approach and policy context. This is consistent with the exploratory and mapping purposes of a scoping review.

**Presentation of the data-charting framework**

**Comment:**

*The data-charting list is extensive and may be clearer if presented in a table.*

**Response:**

 We agree. The data-charting items have been reorganised into a table, grouped under relevant domains. These include study characteristics, characteristics of the health policy decision-maker population, KT intervention characteristics, policy context, evaluation use and theoretical orientation, evaluation approach, evaluation design, data-collection and analytical methods, outcomes, sex- and gender-related reporting, methodological limitations and author reflections.

 This presentation is intended to improve readability and make the planned extraction process more readily reproducible.

**Description of the analysis**

**Comment:**

*The proposed analysis beyond data charting is vague. The terms “synthesised descriptively” and “synthesised narratively” require further explanation and methodological support.*

**Response:**

 We agree and have expanded the Analysis section substantially. The revised section distinguishes among three related stages of analysis.

 First, descriptive numerical summaries will be used to map study characteristics, KT intervention types, populations, contexts, evaluation designs, data-collection methods, outcomes and other categorical variables. Counts will be interpreted as characteristics of the included evidence base rather than estimates of prevalence beyond that evidence base.

 Second, evaluation characteristics will be mapped using the language of the Evaluation Metro Map. This will support consistent consideration of the intended use of the evaluation, its theoretical orientation and its design and methodological characteristics. Explicitly reported information will be distinguished from cautious reviewer interpretation.

 Third, a structured narrative analysis will examine patterns across the included studies, including relationships among intervention aims, intervention complexity, intended outcomes, policy contexts and evaluation choices. The purpose will be to identify recurring patterns, areas of variation and gaps in reporting or evaluative coverage. The analysis will not be used to determine intervention effectiveness or to rank evaluation designs.

 The phrases “synthesised descriptively” and “synthesised narratively” have been replaced with more specific descriptions of the analytical procedures. Supporting methodological references have also been added.

**Scope of the review and suitability of a scoping review**

**Comment:**

*The scope appears large and there are several research questions. A systematic review may be more appropriate if several completed studies are expected.*

**Response:**

 We considered this concern carefully but have retained the scoping review design.

 The primary purpose of the review is to identify and characterise the range of evaluation approaches, designs and methods used in relation to KT interventions involving health policy decision makers. It is not intended to estimate intervention effectiveness, compare a narrowly specified set of interventions or produce a pooled estimate of effect.

 The expected evidence base is heterogeneous in relation to the types of KT intervention, policy setting, intended use, outcomes, evaluation design and data-collection methods. A scoping review is therefore more appropriate for mapping how evaluation has been undertaken and identifying areas in which evidence or methodological reporting is limited.

 The review questions have been reviewed to ensure that they operate as related subquestions under one overarching mapping objective rather than as separate reviews. The objectives and analysis have also been revised to clarify their relationship and to ensure that the proposed work remains feasible within the review’s scope.

 The existence of completed evaluation studies does not in itself require a systematic review of effectiveness. A subsequent systematic review could be warranted if this scoping review identifies a sufficiently coherent group of interventions, outcomes and designs capable of supporting a narrower effectiveness or methodological question.

**Consistency of tense**

**Comment:**

*The manuscript uses a mixture of past, present and future tense and should be revised for consistency.*

**Response:**

 The manuscript has been reviewed for consistency of tense. Procedures that are proposed within the protocol are now described in the future tense. Actions that had already occurred before revision, including development of the original search and its retrospective PRESS assessment, are described in the past or present perfect tense where necessary for transparency.

**Missing references**

**Comment:**

*Several references are missing, including references for PRISMA-ScR and Rayyan.*

**Response:**

 We agree and have conducted a full review of the manuscript’s citations. References have been added or corrected for PRISMA-ScR, Rayyan, the PCC framework, PRESS, the Evaluation Metro Map, the SAGER guidelines and the methodological guidance informing the analysis. Citations relating to the review’s conceptual background and data-charting procedures have also been checked for completeness and accuracy.

# Reviewer report: Reviewer response for version 1

**DOI:** 10.21956/hrbopenres.15790.r54476

**Published:** 2026-05-05

**Authors:** Ian Graham

**Affiliations:** 1 School of Epidemiology and Pubic Health, University of Ottawa Faculty of Medicine (Ringgold ID: 12365), Ottawa, Ontario, Canada; 2 Ottawa Hospital Research Institute, Ottawa, Ontario, Canada

This paper presents a scoping review protocol examining how KT interventions directed at health policymakers are evaluated. The review is timely and will highlight the current evaluation approaches, designs and methods used in evaluating KT interventions involving policymakers. The proposed methodology is appropriate and rigorous. Table 1 providing the conceptual overview is excellent, although the rational for the ordering of the rows is not clear. Starting with the Push pull, exchange strategy in column one and then listing all KT interventions related to each together could help some readers better appreciate similar types of KT strategies and interventions.

 I expect that few studies actually label the evaluation approach taken, but if the information is provided or can be gleaned, using the Lemire (2024) Evaluation Metro Map (eg use line, theory line, design line (which is already mostly incorporated into the review), etc (or another such taxonomy) might be informative. Information on the theory line would provide insight into epistemological perspectives of the evaluations.

 Other minor comments/suggestions for consideration include:

 -It is not clear whether health policymakers are included in the study team.

 -Defining policymakers in the background section would be help as the term is not defined until page 6 under the PCC. Additionally, it is not entirely clear what is the difference between health policymakers and policy-level decisionmakers. Is the intent that the policy-level decision makers refers to those outside of the health sector (eg policymakers in education, justice, finance taking decisions that affect health?) Or is the distinction referring to something else?

 -Including a sex and gender-based analysis of the data might prove interesting. While I expect sex or/and gender of evaluation participants is likely not reported often or at all, reporting this as a finding might encourage others evaluating KT interventions directed at policymakers to consider whether KT interventions work the same way and have the same effect on policymakers of different sex/genders (more likely gender effects than sex effects) and potential sex/gender differences of policymakers’ preferences for KT interventions. If the review collects and reports data on sex/gender, consider using the SAGER reporting guideline.

 -Given the availability of reasonably good AI translation technologies, a rational for only including studies published in English might be warranted.

 -Is there a reason the search strategy was not PRESSed? While mentioned in the abstract, the search strategy section does not state the time period that will be searched.

 -As evaluation approach (Utilization focused evaluation, realist evaluation, equity focused evaluation etc) and evaluation design (case study, ITS, RCT etc) are distinct, there might be value in defining both terms and ensuring they are not used as synonyms in the protocol.

 Best of luck with the review. I am looking forward to reading it soon.

Is the study design appropriate for the research question?

Yes

Is the rationale for, and objectives of, the study clearly described?

Yes

Are sufficient details of the methods provided to allow replication by others?

Yes

Are the datasets clearly presented in a useable and accessible format?

Yes

Reviewer Expertise:

implementation science, KT, KM, health services research

I confirm that I have read this submission and believe that I have an appropriate level of expertise to confirm that it is of an acceptable scientific standard.

## References

[1] : The Evaluation Metro Map. *Journal of MultiDisciplinary Evaluation* .2024;20(48) : 10.56645/jmde.v20i48.106335-40 10.56645/jmde.v20i48.1063

# Response

**Published:** 2026-07-15

**Authors:** Amelia Phillips

**Affiliations:** Nursing and Midwifery, University of Galway, Galway, County Galway, Ireland

We sincerely thank Dr. Graham for his thoughtful and constructive review. His comments were particularly helpful in strengthening the conceptual clarity of the protocol, especially in relation to the distinction between evaluation approach and evaluation design, the consistent definition of the population of interest, and the use of the Evaluation Metro Map. We have given careful consideration to each suggestion.

**General comments**

**Comment:**

*This paper presents a scoping review protocol examining how KT interventions directed at health policymakers are evaluated. The review is timely and will highlight the current evaluation approaches, designs and methods used in evaluating KT interventions involving policymakers. The proposed methodology is appropriate and rigorous.*

**Response:**

 We thank the reviewer for this positive assessment and for recognising the relevance and methodological rigour of the proposed review.

**Organisation of Table 1**

**Comment:**

*Table 1 provides an excellent conceptual overview, although the rationale for the ordering of the rows is unclear. Starting with the push, pull and exchange strategies and then grouping the related KT interventions may help readers appreciate similarities between the strategies and interventions.*

**Response:**

 Thank you, and we do agree. Table 1 has been reorganised so that the principal KT orientations of push, pull and exchange provide the primary structure. Interventions associated with each orientation are now grouped together. Where an intervention may involve more than one orientation, this is acknowledged rather than assigning it unambiguously to a single category. This revision is intended to make the conceptual organisation clearer while recognising that KT interventions may combine multiple strategies.

**Use of the Evaluation Metro Map**

**Comment:**

*Few studies may explicitly label the evaluation approach taken, but where this information is reported or can be inferred, the Lemire Evaluation Metro Map, or another taxonomy, may be informative. The theory line may also provide insight into the epistemological perspectives of the evaluations.*

**Response:**

 We agree and have adopted Lemire’s Evaluation Metro Map as an organising and communication tool within the review.

 The Metro Map will be used to describe relevant dimensions of the evaluations consistently, including their intended use, theoretical orientation and design characteristics. An additional extraction will be undertaken using the language of the Metro Map across all included studies. This will allow the review to communicate more clearly what each evaluation appears to be trying to accomplish, how it is conceptually informed and how it is operationalised.

 We recognise that many studies may not explicitly identify a named evaluation approach or theoretical orientation. We will therefore distinguish between information that is explicitly reported by study authors and information that can be cautiously interpreted from the study description. We will avoid assigning a named approach where there is insufficient evidence to support that classification.

 The Metro Map will not be used as a quality-assessment or ranking tool. It will instead provide a shared language through which the different dimensions of an evaluation can be described without reducing the evaluation to its study design alone.

 A citation to Lemire’s Evaluation Metro Map has been added, and its role in the extraction and analysis has been explained in the Methods section.

 Involvement of health policy decision makers in the review team

**Comment:**

*It is unclear whether health policymakers are included in the study team.*

**Response:**

 No health policy decision makers are formal members of the review team. This will now be stated explicitly in the manuscript.

 There will be health policymakers included at the end of this study to assist with dissemination and translation of research findings. In all subsequent studies for the PhD project there will be consistent health policymaker inclusion. It would have been preferable to have policymaker input from the conception of this review, however there was time needed to build networks with the relevant individuals and that overlapped with the review timeline.

**Definition and consistency of terminology**

**Comment:**

*Policymakers should be defined earlier in the manuscript. It is also unclear how “health policymakers” differ from “policy-level decision makers”.*

**Response:**

 We agree that the previous terminology introduced unnecessary ambiguity. The manuscript has been revised to use the term health policy decision makers consistently and exclusively when referring to the population of interest.

 A definition has been added to the Background section before the term is used in the eligibility criteria. For the purposes of this review, health policy decision makers are defined as individuals who have a formal or substantive role in developing, influencing, implementing, commissioning or advising on decisions relating to health policy, health services or health systems.

 This may include decision makers based within government departments, public health agencies, health service organisations, regulatory bodies and other organisations with responsibility for health policy decisions. Individuals working outside the health sector will be included only where their role involves identifiable decisions that directly affect health policy, population health or health-system organisation.

 The term “policy-level decision makers” has been removed to avoid suggesting that it represents a separate population.

**Sex- and gender-related data**

**Comment:**

*The review should consider collecting and reporting sex- and gender-related data. Even if these characteristics are rarely reported, their absence may be an important finding. The SAGER reporting guideline should also be considered.*

**Response:**

 We agree. The data-charting framework has been amended to collect information on the sex and/or gender of evaluation participants where this is reported in the included studies.

 We will record the terminology used by the original authors and will not treat sex and gender as interchangeable or infer either characteristic where it has not been reported. Where studies provide data that allow findings to be examined by sex or gender, this will also be recorded. However, we anticipate that the extent of reporting may be limited.

 The review will therefore report both the information provided and the extent to which sex and gender are absent from evaluation reporting. The implications of limited reporting for understanding whether KT interventions may be experienced differently by health policy decision makers of different sexes or genders will be discussed cautiously.

 The SAGER guidelines have been added as a reporting reference and will inform how these data and their limitations are presented.

**English-language restriction**

**Comment:**

*Given the availability of reasonably good AI translation technologies, a rationale should be provided for including only studies published in English.*

**Response:**

 We agree that developments in machine-assisted and AI-supported translation have increased the potential accessibility of studies published in other languages. However, the English-language restriction will be retained as a feasibility constraint and will be justified more clearly in the manuscript.

 Although AI-generated translation may support initial comprehension, translations of technical methodological descriptions, theoretical concepts and context-specific policy terminology would still require verification to ensure that the extracted information was accurate. This would require access to reviewers or translators with appropriate language proficiency and subject knowledge.

 The review includes detailed extraction relating to evaluation theory, rationale, design, methods, outcomes and policy context. Inaccuracies in the translation of these concepts could materially affect study classification and interpretation. Verifying translations across potentially multiple languages would therefore introduce substantial additional work that cannot be accommodated within the timeframe and resources of the PhD.

 The manuscript will acknowledge that the restriction may exclude relevant evidence and may limit the geographical breadth of the review.

**PRESS review and search dates**

**Comment:**

*The reason the search strategy was not PRESS reviewed is unclear. The search period should also be stated in the search strategy section.*

**Response:**

 We agree that this requires further explanation. The original search strategy was not prospectively peer reviewed using the PRESS framework because of the timing and resource constraints associated with conducting the review within the PhD programme.

 A retrospective PRESS review of the search strategy is now being undertaken by an information specialist at the University of Galway. So far, the PRESS review has recommended using Ovid MEDLINE ALL rather than PubMed for any revised search, presenting the strategy line by line with hit counts, combining database-specific subject headings with free-text terms, and reviewing spelling variants, proximity operators and evaluation-related terminology. It also prompted consideration of overlap between ScienceDirect and Scopus.

 The manuscript will state clearly that the PRESS assessment was retrospective rather than implying that it occurred before the searches were conducted. Any issues identified through the retrospective review, and any resulting amendments or implications for the review, will be documented transparently.

 The Methods section will also be revised to state explicitly that the search covers literature published from 2000 to the date on which each database search was conducted. The final search date for each database will be reported.

**Distinction between evaluation approach and evaluation design**

**Comment:**

*Evaluation approach and evaluation design are distinct concepts and should be defined and not used as synonyms.*

**Response:**

 We agree and have revised the manuscript to distinguish these concepts consistently.

 An evaluation approach refers to the broader orientation or set of principles that shapes how an evaluation is understood and undertaken. Examples may include realist, utilisation-focused, developmental or equity-focused evaluation.

 An evaluation design refers to the empirical structure used to generate and analyse evidence, such as a case study, randomised controlled trial, interrupted time-series study, before-and-after study or qualitative interview study.

 The previous manuscript did not always communicate this distinction sufficiently clearly. The relevant sections have therefore been revised to ensure that evaluation approach, design, methods and intended use are not treated as interchangeable concepts.

 Adoption of the Evaluation Metro Map will support this distinction by providing language through which the different dimensions of an evaluation can be described separately. The review will not infer that a study’s design, such as a case study or randomised trial, constitutes its broader evaluation approach. Where an approach is not named or cannot be identified with reasonable confidence, this will be reported as not stated or unclear rather than being derived solely from the design.
